# Reward-related activity in the medial prefrontal cortex is driven by consumption

**DOI:** 10.3389/fnins.2013.00056

**Published:** 2013-04-11

**Authors:** Nicole K. Horst, Mark Laubach

**Affiliations:** ^1^The John B. Pierce LaboratoryNew Haven, CT, USA; ^2^Interdepartmental Neuroscience Program, Yale University School of MedicineNew Haven, CT, USA; ^3^Department of Neurobiology, Yale University School of MedicineNew Haven, CT, USA

**Keywords:** feeding, field potential, food, foraging, licking, phase locking, population activity, theta

## Abstract

An emerging literature suggests that the medial prefrontal cortex (mPFC) is crucial for the ability to track behavioral outcomes over time and has a critical role in successful foraging. Here, we examine this issue by analyzing changes in neuronal spike activity and local field potentials in the rat mPFC in relation to the consumption of rewarding stimuli. Using multi-electrode recording methods, we simultaneously recorded from ensembles of neurons and field potentials in the mPFC during the performance of an operant-delayed alternation task and a variable-interval licking procedure. In both tasks, we found that consummatory behavior (licking) activates many mPFC neurons and is associated with theta-band phase locking by mPFC field potentials. Many neurons that were modulated by the delivery of reward were also modulated when rats emitted bouts of licks during the period of consumption. The majority of these licking-modulated neurons were found in the rostral part of the prelimbic cortex, a region that is heavily interconnected with the gustatory insular cortex and projects to subcortical feeding-related centers. Based on the tight coupling between spike activity, theta-band phase locking, and licking behavior, we suggest that reward-related activity in the mPFC is driven by consummatory behavior.

## Introduction

Successful foraging requires exploration of the surrounding environment, the capacity to remember where food/water was previously encountered, and the flexibility to adapt behavior in response to depleting resources or when confronted with competitors or predators (Stephens and Krebs, 1986). The tendency for animals to spontaneously alternate responding between multiple available locations over short time spans has been observed in numerous mammalian species, including rodents, and is considered to be an innate foraging strategy that allows animals to efficiently and thoroughly sample their environment (Estes and Schoeffler, 1955; Whishaw et al., 1995; Olton and Samuelson, 1976). Laboratory studies using mazes (such as a T-maze or radial arm maze) or operant procedures have revealed that this behavior depends upon functional medial prefrontal cortex (mPFC) for working memory processes that track which location to visit, along with inhibitory processes that prevent the animal from revisiting a particular location on adjacent trials (Seamans et al., 1995; Floresco et al., 1997; Horst and Laubach, 2009).

Neuronal recordings have revealed the mPFC neurons respond to many aspects of behavioral performance, including choice location, whether or not the trial was performed successfully, and time since the previous response (e.g., Horst and Laubach, 2012). More complex tasks have confirmed a role for medial regions of the PFC in foraging behaviors. Neuronal activity in the rat anterior cingulate cortex (ACC), posterior to the mPFC region studied in the Horst and Laubach study, reflects cost-benefit calculations based on the presence or absence of a competitor (Hillman and Bilkey, 2012; reviewed by Walton and Baudonnat, 2012). In monkey ACC, neurons reflect the relative cost of staying in a patch (a location with depleting food) vs. switching to a new patch (Hayden et al., 2011). A recent human study reported ACC activity being greater when participants search for alternative options, and ventromedial PFC activity corresponds better with the final decision (Kolling et al., 2012). Each of these examples requires not only information gathering about the potential response options (locations), but also the ability to detect and evaluate the outcome (reward).

Such processes might depend on the ability of the mPFC to integrate behavioral outcomes over time. Several recent studies have reported neurons in the mPFC that track outcomes from one trial to the next (e.g., Narayanan and Laubach, 2008; Hayden et al., 2009; Horst and Laubach, 2012; Hyman et al., 2012). A major open question is whether mPFC neurons are driven by some abstract “outcome-related” signal or by an observable behavior associated with reward consumption. This issue can be addressed in rodent studies by measuring the precise timing of reward delivery and consummatory behavior (licking measured with a photobeam detector) and relating these measures to neuronal spike activity and field potentials in the mPFC. Previous neuronal recording studies have reported that mPFC neurons are modulated differentially in anticipation of and by the receipt of different types of rewards (e.g., food vs. water; Pratt and Mizumori, 2001; Miyazaki et al., 2004; Petykó et al., 2009), in anticipation of different amounts of reward (Kargo et al., 2007), and by both liquid rewards and rewarding electrical brain stimulation (Takenouchi et al., 1999). Only one of these studies measured the precise timing of licking and found no correspondence between spikes and licks in the mPFC (Takenouchi et al., 1999). Importantly, the recordings in this study were made in the peri-genual ACC region of the rodent mPFC.

In the present study, we evaluated the role of the rat mPFC in reward processing during two tasks: an operant-delayed alternation task that depends on working memory and is disrupted following inactivation of the mPFC (Horst and Laubach, 2009) and a variable-interval licking procedure that simply required rats to stand at a spout and lick until fluid was delivered. We recorded single unit activity and field potential signals in separate cohorts of rats performing the two tasks. (The recordings were made mostly in the prelimbic area, rostral to the recordings in the Takenouchi study cited above.) We identified neurons that were modulated by either the receipt of reward, the initiation of reward consumption, or both of these factors. By using the same methods as Gutierrez et al. (2006); Gutierrez et al. (2010), we identified persistent bouts of licking and found evidence for changes in neuronal activity around bouts that occurred after the initial receipt of reward. We also examined the anatomical distribution of neurons with licking-related sensitivity. Our findings suggest that reward-related activity, especially in rostral mPFC, can be driven by consummatory behavior.

## Materials and methods

All rats were handled daily and had free access to food and water for one week upon arrival in the laboratory. Rats had regulated access to water for 1 week prior to training and throughout the period of training and testing. Food was available *ad libitum.* During training sessions, rats were reinforced with water, which was supplemented in the home cage to maintain them at ~90% of their free access body weight. All procedures were approved by the Animal Care and Use Committee at the John B. Pierce Laboratory and conformed to the standards of the United States National Institutes of Health Guide for the Care and Use of Laboratory Animals. All efforts were taken to minimize the number of animals used and to reduce pain and suffering.

### Behavioral tasks

#### Operant-delayed alteration task

**Figure 1:**
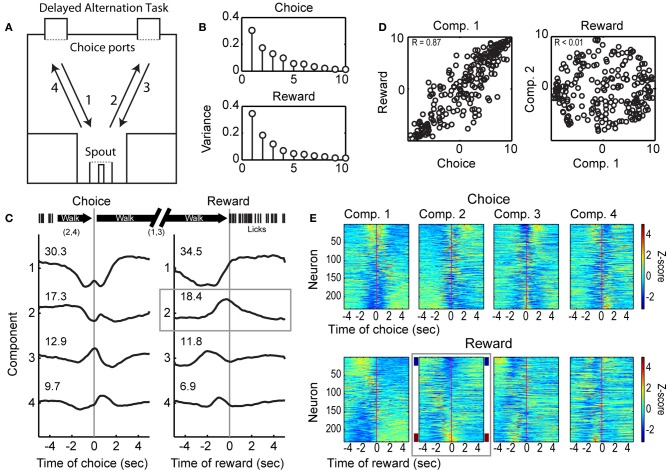
**Choice- and reward-related modulations of spike activity in the delayed alternation task. (A)** Design of the operant-delayed alternation task. Rats responded at two “choice ports” located on one side (top) of an operant arena. They received water from a spout, located on the opposite side of the arena (bottom), if they responded in an alternating manner at the choice ports (e.g., following arrows 1–4). **(B)** Temporal patterns of population activity from mPFC were quantified using PCA across the 249 neurons recorded across 5 rats (Narayanan and Laubach, 2009). The variances associated with the 10 leading Components based on activity around the choice and reward events are shown. **(C)** Population activity associated with the four leading Components for the choice and reward events. The first Components for each event were dominated by fluctuations between periods of locomotion. The second Component for the reward event was associated specifically with modulation in firing rate around the time when rats received water (highlighted by the gray box). **(D)** Scatter plots of loadings (weights) for each neuron on the leading Components for the choice and reward task events are shown on the left. Neurons had similar weights on the Components across the task events (*R* = 0.87). Loadings for the first and second Component for the reward event were orthogonal (*R* < 0.01). **(E)** Spike activity associated with the leading Components for the choice and reward events. Firing rates were normalized using Z-scores and sorted by the loadings of each neuron onto the Components. Firing rate modulations around reward receipt were apparent for Component 2 (highlighted by the gray box), with some neurons firing at higher rates (~10% with *Z* < −2, maroon sideband) and other neurons firing at lower rates (~10% with *Z* > 2, navy sideband) when rats arrived at the spout and consumed the reward.

Long-Evans (*N* = 5) or Brown Norway (*N* = 4) rats (3–6 mo old, male) were trained to perform an operant-delayed alternation procedure that depends upon mPFC function (Horst and Laubach, 2009, 2012; Caetano et al., 2012a,b). Rats were trained in an operant box (ENV-008, Med Associates) housed within a sound-attenuating chamber (ENV-022V). A spout positioned between two barriers (which controlled for the posture of rats during reward consumption), was located at one end of the chamber and two choice ports were located on the opposite wall (Figure 1A). All response locations were equipped with infrared beams that detected head entries into the choice ports and licks to the reward spouts. All response devices were custom-made by the Instruments Shop at the John B. Pierce Laboratory and were controlled using software (MedPC) and a computer interface from MedAssociates.

Rats were trained as previously described (Horst and Laubach, 2009, 2012). Fully trained rats were required to alternate their choices between left and right ports in order to receive water reward (delivered in six 0.25 s pulses separated by 0.50 s pauses or constant over 1.0 s at 0.05 ml per s) on the opposite side of the operant box. An example of a successful alternation trial is shown in Figure 1A, following arrows 1–4. After a correct response in the left choice port, rats would cross the box to collect reward (1), then alternate to the right choice port (2), for another reward (3). The rat would then alternate back to the left choice port (4). If the same port [e.g., a rightward response after (3) above] was chosen on consecutive trials, it was scored as an error, the lights in the chamber were extinguished, and the rat had to contact the spout before making the next choice (although no reward was delivered). After an error, the target location for the correct choice was always the choice port opposite to the one chosen erroneously. Behavioral and neurophysiological data related to working memory performance in this task have been published previously (Horst and Laubach, 2012). This paper focuses on the modulation of neurons by consumption-related behaviors, which have not been described previously in the mPFC of rats performing a working memory task.

#### Variable-interval licking procedure

Prior to any behavioral training, four adult male Long-Evans rats were implanted with multielectrode arrays (see surgical methods below). After at least 1 week of recovery from surgery, rats were placed in the operant box with a spout positioned between two barriers and no access to the choice ports (Figure 6A). Presentation of an auditory click stimulus and illumination of a light over the reward port on a variable-interval schedule signaled the availability of water (0.04 ml per s for 3 s) (see Figure 6B, top panel, for interval distribution from a sample session). Delivery of water was triggered by the first lick captured after presentation of the auditory and visual cues. Animals tended to remain near the spout after the first training session, and licked on the spout for prolonged periods of time. Rats were trained in the variable-interval procedure for 10 days. At this point, rats should have learned to anticipate the water around the mean interval, based on Kirkpatrick and Church (2003) who used 320 reinforcements as a criterion for temporal learning. This criterion was achieved for all rats in no more than 4 (3.67 ± 0.58) sessions, and analysis of the neuronal results was made in the final testing session.

### Surgeries

Twelve rats were implanted with fixed arrays made using 50 μm stainless steel wire, which had *in vitro* impedance between 200 and 300 kΩ, arranged in a 2 × 8 configuration with ~200 μm between electrodes and the arrays spanning 2 mm in total (NB Labs). One rat was implanted with a drivable array of microelectrodes (1 × 8 configuration, CD Neural Technologies). Rats in the delayed alternation task were trained until they performed with accuracy greater than 75% correct, as described previously (Horst and Laubach, 2012). They were then given at least 1 week of full access to food and water and implanted with microwire arrays into mPFC. Rats trained in the variable-interval task were implanted before experiencing any behavioral testing.

After initial anesthesia with ~4% halothane, intraperitoneal injections of ketamine (80–1.0 mg/kg) and diazepam (8–10 mg/kg), or ketamine and xylazine (10 mg/kg) were administered. Supplements (1/3 of the initial dose) of the two drugs administered in the procedure were given approximately every 60 min. Using standard methods, arrays of microwire electrodes were placed in mPFC using a craniotomy that was centered at 3.2 mm rostral to bregma and ±1.4 mm lateral to bregma. The arrays were placed to avoid major vessels within the craniotomy. Fixed 2 × 8 arrays were lowered to a depth of 3.7 mm at an angle of 12° from the midline, placing the electrode tips at ~3.5 mm ventral to the skull and 0.5 mm from the midline. The microdrive was placed at the following coordinates: AP: +3.2, ML: +1.4, DV: −2.8 mm at 12° from the midline. The microdrive was lowered in steps of 0.05 mm every day throughout the period of the recordings (45 days).

Of the nine rats trained in the spatial delayed alternation task, one became ill, one did not complete any trials after microelectrode implantation, and two did not have clear single unit activity. One of the rats in the variable-interval task did not have clear single units. Data from these five rats were excluded from all aspects of the study.

### Electrophysiological recordings

One week after surgery, neuronal recordings were made during behavioral sessions using a multi-electrode recording system (Plexon). Single units were identified on-line using an oscilloscope and audio monitor and off-line using the Plexon off-line sorter. On-line sorting was done with the “boxes” feature in the Plexon software, in which waveforms were manually selected based on their amplitude and deviation from background firing. Artifacts due to cable noise and behavioral devices were removed during off-line sorting. Single units were identified as having (a) consistent waveform shape, (b) average amplitude estimated at least three times larger than background activity, and (c) a consistent refractory period of at least 2 ms in interspike interval histograms. Behavioral events in the task were streamed to the Plexon on-line sorter via TTL pulses delivered from the MedAssociates system. This allowed the neuronal data to be accurately synchronized to these events.

Field potentials were sampled on all electrodes and recorded continuously throughout the behavioral testing sessions using the Plexon system. The sampling rate was 1 kHz. The head-stage filter (Plexon) was between 0.5 and 5.9 kHz. Electrodes with unstable signals (assessed using the 1D Viewer plotting function in NeuroExplorer), clear peaks at 60 Hz in power spectral density functions, plotted as percent total power spectrum in NeuroExplorer, that were based on recordings over the entire behavioral session, or with large spike waveforms recorded on the electrode were excluded from quantitative analysis. The animal with the drivable microelectrode array was excluded from this analysis due to the microdrive using 15 μm wires that integrated signals over much smaller cortical volumes. Power spectra were not equivalent between LFP recordings from the microdrive and chronic arrays.

### Histological procedures

Once experiments were complete, rats were anesthetized with 100 mg/kg sodium pentobarbital and perfused with 10% formalin. Brains were sectioned horizontally on a freezing microtome, mounted on subbed slides, and stained for Nissl substance with thionin. Histological examination of electrode tracts found that recording sites in all rats were within the mPFC (see Horst and Laubach, 2012 and Figure 8).

### Data analyses

Exploratory analyses of neuronal activity and behavior were performed with NeuroExplorer (Nex Technologies). Statistical analyses of neuronal and behavioral data were performed using Matlab (Mathworks) and R (www.r-project.org). Computer code used for the data analysis in the study is available upon request from the corresponding author.

#### Analysis of population activity around reward collection

Temporal variability over the neuronal population was assessed using Principal Component Analysis (PCA), using methods from Narayanan and Laubach (2009). The method finds common sources of variance in the temporal patterns of spike discharge over the population of neurons. The activity of each single neuron was summarized as a trial-averaged spike-density function (similar to a peri-event time histogram; bin size of 0.05 s) centered around the time of the spatial choice, the time of reward delivery, or the onset of a bout of licks. For each task event, a data matrix was assembled that contained spike-density functions (normalized to have a mean of 0 and variance of 1, i.e., z-score function in Matlab) in each row (J) for each neuron and the columns represented time around the event of interest. Eigenanalysis was done for N leading Components in Matlab as:

  [u,s,v] = svd(X',0); U = u(:,1:N);
  l = diag(s).^2/(J-1); Z = X^*^U;


Here, U contains the eigenvectors (or coefficients) for each bin, l holds the eigenvalues (or variance explained), and Z is the matrix of scores (or loadings) for the neurons onto each Component. The transpose is used (e.g., X′) to account for variability over bins and not neurons. The coefficients for each Component function described the temporal variation in firing rates around the event of interest. The loadings (or scores) for each neuron accounted for the temporal similarity between the firing rate of a given Component.

To assess temporal relationships between the leading Components, we plotted each Component against cumulative sums (cusum in Matlab) of the coefficients for other Components (as in Narayanan and Laubach, 2009). For the plots shown in Figures 4D and 6E, we scaled the cumulative sums by subtracting their overall average value, dividing by the maximum absolute value of the residual signal, and multiplying by the maximum absolute value signal of the coefficients for the raw Components.

#### Analysis of lick-related spike activity

**Figure 2:**
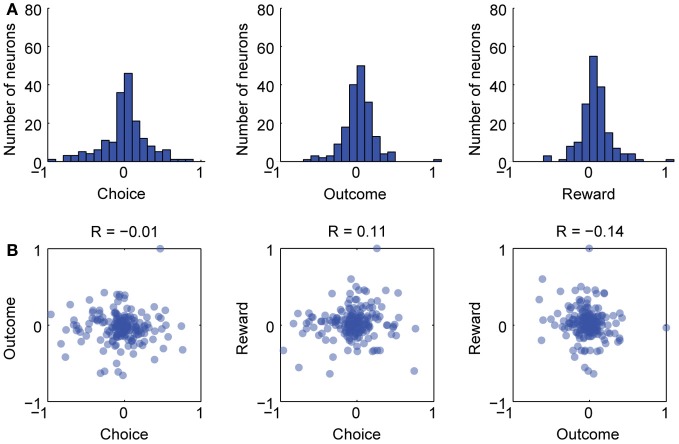
**Separate groups of neurons varied with choice, outcome, and reward in the delayed alternation task. (A)** Firing rate indices were calculated for choice, outcome, and reward delivery (see text for details) and histograms for these are shown. **(B)** Scatterplots for the three indices. There was no clear linear relationship between the indices over neurons. Therefore, there were few, if any, neurons that covaried across choice, outcome, and reward delivery. Pearson correlation coefficients (*R*-values) are given for each scatterplot and none were significant at *p* < 0.05.

Modulations of spike counts around choice, outcome feedback, initiation of licking, and reward delivery and from the onset and offset of bouts of licks were compared across windows of 200 ms before and after the event using signed-rank tests, with a criterion of *p* < 0.05 based on permutation test (1000 iterations per neuron). The use of 200 ms bins captured the peak inter-trial coherence from the field potential analysis and was associated with maximal firing rates in neurons that showed clear shifts in activity around onsets of licking and/or reward delivery in our exploratory data analysis. The first lick in each bout was identified and used to synchronize spike activity around licking bouts. To examine whether the same neurons encoded information about choice, feedback about the outcome of the choice, and reward delivery, we calculated a modulation score using spike counts [e.g., modulation score for Choice = (SC_left_ − SC_right_)/(SC_left_ + SC_right_)] for each neuron for each of these events. In the example provided, a negative score would indicate greater firing for a left vs. right choice, and vice versa for a positive score, with larger numerical values corresponding to greater modulation. The scores for each neuron were plotted to determine whether neurons that were modulated by one event tended to be influenced by other events (Figure 2B). The fractions of cells that showed modulation around licking initiation vs. reward delivery and bout onsets vs. offsets were compared using a Chi-square proportions test.

#### Analysis of local field potentials

Spectral analysis of field potentials used methods from the EEGLab toolbox for Matlab (Delorme and Makeig, 2004). A peri-event matrix (FPmat) was created to examine modulations around reward delivery, first lick, or bouts of licks using 1 ms bins for the epoch ±2 s around each event. The sampling rate was thus 1 kHz. Rows in the matrix contained the trials. Columns contained the samples (bins). The matrix was multiplied by 1000 to convert from millivolts (Plexon files) to microvolts (assumed by EEGLab). Trials were excluded from the analysis if they contained values greater than four times the standard deviation of the absolute voltage level, i.e., in Matlab: std(abs(FPmat(:)))^*^4. Event-related spectral amplitude and phase were calculated using the function *newtimef* from EEGLab:

   [ERSP, ITC, ~, times, freqs] = newtimef
   (FPmat, 4000, [−2000 2000], 1000, [1 4],
   ‘freqs’, [1 50], ‘nfreqs’, 50, ‘baseline’,
   NaN, ‘plotphasesign’, ‘off’);


This function returned the Event-Related Spectral Power (ERSP) and the Inter-Trial Coherence (ITC) (or “phase-locking factor”), which reflects the degree of coherence in the trial-by-trial distribution of phase angles over the frequency bands, ranging from 1 to 50 Hz.

The analysis involved measuring for each trial the instantaneous amplitude and phase of the field potential for each step in the frequency axis. The ERSP matrix contained the power at each time and frequency, calculated for each field as:

$$\text{ERSP}\left(f,t\right)=\frac{\text{1}}{n}\sum_{k\;=\text{ 1}}^{n}{\left|F_{k}\left(f,t\right)\right|}^{\text{2}}$$

where *F*_*k*_(*f, t*) is the spectral estimate of amplitude for trial *k* at frequency *f* and time *t* (Delorme and Makeig, 2004). Prior to averaging over trials, the mean field potential (Event-Related Potential) was removed from each trial (i.e., baseline correction involved removing the average for the entire task epoch). Power was then calculated by conversion to a decibel (dB) scale (10^*^log10[power(*t*)/power(baseline)]). This allowed for direct comparisons of power across frequencies.

The ITC matrix contained the coherence (correlation in the frequency domain) between the phase angles of a given field potential at each time and frequency. ITC was defined as:

$$\text{ITC}\left(f,t\right)=\frac{\text{1}}{n}\sum_{k\;=\text{ 1}}^{n}\frac{F_{k}\left(f,t\right)}{\left|F_{k}\left(f,t\right)\right|}$$

where *F*_*k*_(*f, t*) is the spectral estimate of phase for trial *k* at frequency *f* and time *t* (Delorme and Makeig, 2004). The ITC is an average measure and ranges from 0 to 1. An ITC value of 0 indicates random phases over trials and a value of 1 indicates identical phases over trials. Analysis was done using standard FFT-based methods (cycles = 0) and using Morlet wavelets (a Gaussian-shaped window in the frequency domain) with various parameters for the number of cycles in each data window. The wavelet methods proved better at capturing transient events. For the results shown in the paper, we used Morlet wavelets with increasing cycles (from 1 to 4) over the frequency space (from 1 to 50 Hz).

Significant fluctuations in power and phase were assessed using a bootstrap method. A surrogate data set was constructed from randomly selected latency windows in each trial, and then averaging over the selected windows. Using a criterion of *p* < 0.01 resulted in 1000 such surrogates being generated for each field potential, and percentiles were measured from these surrogate distributions to estimate significance thresholds.

**Figure 3:**
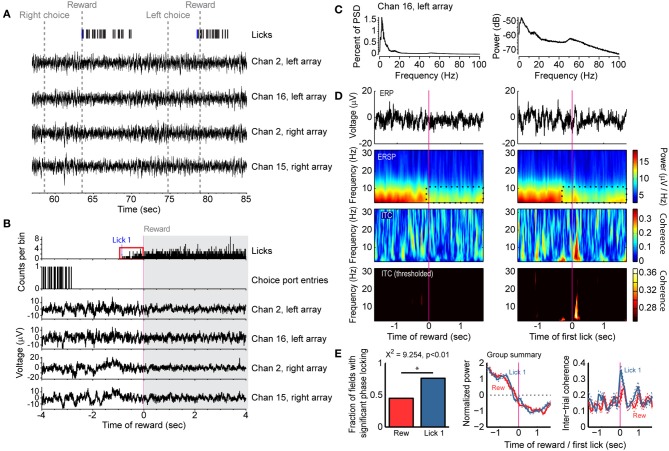
**Reward- and consumption-related modulations of field potentials in the delayed alternation task. (A)** Example of task events and field potentials over two trials. Four field potentials are shown and the first lick accompanying the reward epoch (“Lick 1”) is designated by a blue tick mark. **(B)** Histograms for licks and choice port entries and averaged field potentials for the same four recordings as in **(A)**. Note that rats could lick before receiving reward (red box, allowing for dissociating licking from reward delivery) and that the field potentials showed reduced fluctuations during licking. **(C)** Power spectral density is shown for one field potential from (**A** and **B**), plotted as percent of total power (left) and using a log scale (right). **(D)** Field potentials showed increased power and phase locking around initiation of licking compared to the receipt of reward. Averaged event-related potentials (ERP) are shown in the top row. Event-related spectral power (ERSP) is shown in the second row, and a region with reduced power during reward consumption is highlighted by the dashed black lines. Inter-trial phase coherence (ITC) is shown in the lower two rows. The color scale was thresholded in the lower row to only show levels of inter-trial coherence that were greater than expected by chance. **(E)** Group summary of inter-trial coherence for 54 field potentials recorded in single sessions from 4 rats. The plot denotes the finding that significantly more fields were phase locked [based on the ITC values shown in panel **(D)** at time 0 being above the level obtained from a bootstrap analysis] at the time of licking (Lick 1) compared to the time of reward delivery (Rew) (Chi-square test, *p* < 0.01). The middle plot shows the average spectral power around the time of reward (red) and first lick (blue), and the 95% confidence bands (dashed lines). The right plot shows the average Inter-Trial Coherence for the time of reward (red) and first lick (blue), again with the 95% confidence bands (dashed lines).

For group summaries, inter-trial coherence in the range of delta (1–4 Hz) and theta (4–8 Hz) was extracted from the ITC matrices from each field potential and was used to create group summaries. Confidence intervals were calculated for group data using the *bootci* function from the Matlab Statistics Toolbox. These lower frequency ranges were clearly apparent in power spectra calculated from the field potentials (Figure 3C) and effects related to the task events were apparent in time-frequency maps, as in Figure 3D, without the need for any normalization.

#### Analysis of the microstructure of licking

The experimental setup permitted the collection of individual licks on the reward spout, based on measurements by a photobeam across the tip of the spout and just in front of the space filled by drops of fluid that were emitted from the spout. Bouts of licks were isolated using Matlab code that was kindly provided by Dr. Ranier Gutierrez and used in Gutierrez et al. (2006). Bouts of licks were defined as having at least 3 licks over epochs of at least 0.5 s and have an inter-bout epoch of at least 0.5 s. Please note that bouts that were coincident with rats first receiving reward in the delayed alternation task were removed from the list of identified bouts, so as to eliminate the influences of locomotion and reward delivery *per se* on putative bout-related neuronal activity.

#### Anatomical localization of theta phase-locking in the mPFC

To explore the anatomical specificity of phase-locking during licking behaviors, we divided the mPFC into three regions that were equally spaced in the antero-posterior direction, counted the fields that showed significant and insignificant phase locking (based on the Inter-Trial Coherence statistic) and used Pearson's Chi-Squared test to evaluate differences in the proportion of neurons with theta phase-locking across these regions.

## Results

### Population activity during choice and reward consumption in the delayed alternation task

A total of 249 neurons were isolated and recorded across the five rats performing the delayed alternation procedure. We have previously reported that the largest fraction of event-modulated neurons in mPFC are sensitive to behavioral outcome (correct vs. error) in the delayed alternation task (Horst and Laubach, 2012). Roughly a third of all neurons encoded outcome around the period of reward delivery. To identify common patterns of firing rate modulation during spatial choice and reward collection, we used PCA, as in Narayanan and Laubach (2009). Application of this approach found four leading Components that together account for over 70% of the observed spiking variance (Figure 1B; Components 1–4 shown in Figure 1C). Loadings from the first Component, which accounted for over 30% of the variance around both choice and reward collection, were similar across both task events (Choice vs. Reward, *R* = 0.87, left panel of Figure 1D), and may have been driven by changes in the state of locomotion, i.e., stopping to make a choice before locomoting to the reward port (Component 1) or stopping to collect reward (Component 2; see timeline above Figure 1C). The second Component showed a distinct pattern of variance around the time of reward collection and accounted for nearly 20% of the temporal variance in neuronal activity (Figure 1C, gray box). The loadings of neurons on the first two Components around the reward were orthogonal (Figure 1D, right panel), and therefore likely to be driven by different behavioral processes. Roughly 20% of variance in neuronal activity was driven by reward delivery and/or the onset of licking. Summaries of spike activity associated with the leading Components for choice (top four panels) and reward (lower four panels) are shown in Figure 1E. Firing rates were normalized using Z-scores and are sorted based on their loading onto their respective Principal Components. The panel highlighted with a gray box shows neurons that reduce (~10% with *Z* > 2, navy sidebands) or increase (~10% with *Z* < −2, maroon sidebands) firing rates around reward receipt. These data establish that a significant proportion of the neuronal population is modulated around reward delivery.

### Commingling of choice, outcome feedback, and reward in the delayed alternation task

To assess if the reward modulated neurons were also sensitive to the previous spatial choice made by the rats and the accuracy of that choice, we calculated firing rate indices for choice, outcome, and reward delivery and examined correlations between the indices. The index for choice was defined as (SC_Left_ – SC_Right_)/(SC_Left_ + SC_Right_). The index for outcome was defined as (SC_Correct_ – SC_Incorrect_)/(SC_Correct_ – SC_Incorrect_). The index for reward delivery was defined as (SC_Post_ – SC_Pre_)/(SC_Post_ + SC_Post_). Histograms showing the indices are plotted in Figure 2A. As shown in the scatterplots in Figure 2B, very few cells were strongly modulated by the location of the choice and the outcome feedback (left most plot in Figure 2B). Likewise, very few cells were strongly modulated around reward delivery and also by either choice or outcome feedback (middle and right plots in Figure 2B). These results are similar to Horst and Laubach (2012), especially Figure 8, and add further support to the idea that aspects of the delayed alternation task are encoded by segregated groups of mPFC neurons.

### Theta-range fluctuations track initiation of licking in the delayed alternation task

To examine if the changes in population activity shown in Figure 1 occurred together with changes in broader aspects of network activity, we analyzed 54 field potentials from four rats (excluding the microdrive animal) that were simultaneously recorded with the spike activity and behavioral events (Figure 3). We also explored evaluated whether changes in activity reward delivery were driven explicitly by the arrival of reward or by the consummatory behaviors that would accompany it. As reward spout contact was required to trigger reward delivery, the onset of licking behavior (“first lick” or “Lick 1” in Figure 3) could be dissociated from reward delivery. Power spectra calculated from the fields had clear peaks in the delta (2–4 Hz) and theta (4–8 Hz) ranges (Figure 3C, plotted as percent of total power on left and log scale on right), and fields were dynamically modulated during travel to the spout and during initiation of fluid intake (Figures 3A,B). The specific relationship of the fields to the onset of licking and reward delivery was apparent in event-related spectrograms aligned to the time of reward onset and of initial spout contact (first lick) (Figure 3D). There was increased power and phase-locking (coherence) around initiation of licking but not receipt of reward (Figures 3D,E). However, not all fields showed phase locking around the onset of licking (left plot in Figure 3E). Nevertheless, more fields showed phase locking around the initiation of licking as compared to the time of reward delivery (76.4 vs. 45.0%, χ^2^ = 9.254, *p* < 0.01). These results suggest that reward-related network activity in mPFC is better coupled to the act of consumption than the delivery of reward.

### mPFC neurons are modulated around bouts of licking in the delayed alternation task

**Figure 4:**
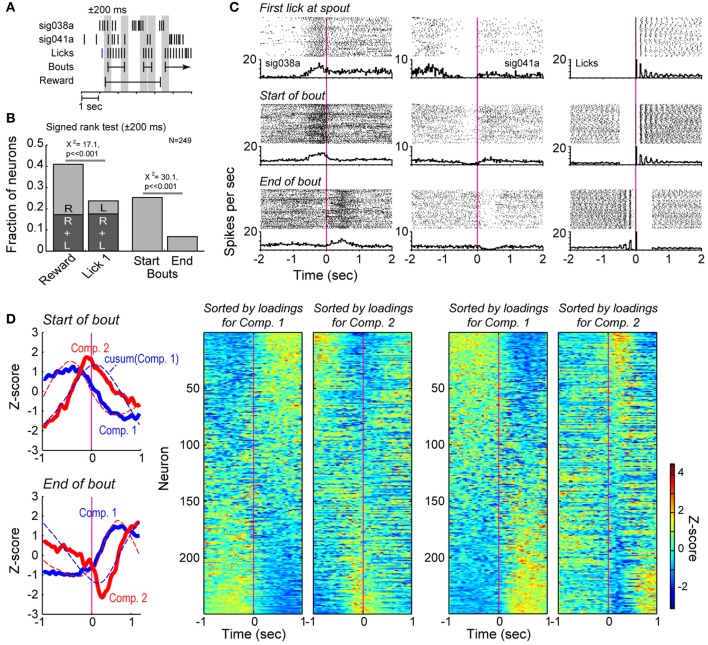
**Bout-related modulations of spike activity in the delayed alternation task. (A)** Example of the data windows (±200 ms) used to assess changes in firing rates around reward delivery and the onset of licking and around bouts of licks. **(B)** Results from signed rank tests of firing rates around reward delivery (R), onset of licking (L), and the onset and offset of bouts of licking. **(C)** Two exemplar neurons that showed significant changes in firing rates when rats first licked for reward and emitted bouts of licks. Raster plots and peri-event histograms are shown for the lick events in the right column. **(D)** Population analysis around the onsets and offsets of the bouts. Two major sources of correlated firing (solid lines in the left plots) varied around the bouts of licking. The first and second Components for bout onsets accounted for 29.3 and 16.1% of variance. The first and second Components for bout offsets accounted for 34.7 and 13.7% of variance. The two cells shown in **(B)** has large, positive loadings on the two Components. Dashed lines in these plots represent the cumulative sums for each Component. Firing rate modulations (as in Figure 1E) are shown for the full population in the matrix plots on the right side of the panel and highlight the major changes in firing rates found over many neurons when rats initiated and terminated bouts of licking.

Fluid consumption by rodents follows a well-defined pattern of activity, with individual licks organized into bouts (Davis and Smith, 1992). To examine how licking behavior influenced spike activity in the mPFC, we used published methods for identifying bouts of licks (Gutierrez et al., 2006) and non-parametric signed rank tests to compare spike counts in 200 ms windows around the onset and offset times of the bouts (Figures 4A,B). We also examined the fractions of neurons with significant modulation (by signed rank test) around reward delivery and the onset of licking after the rats traveled from the choice ports to the spout. (Note that licking onset is measured as the first lick that occurs after a spatial choice, and is not included in the bout analysis.) Nearly half of the neuronal population was sensitive to the either reward delivery or the first lick (Figure 4B). More neurons were sensitive to the delivery of reward (40.9%) than were modulated by the onset of licking (23.6%; χ^2^ = 17.1, *p* << 0.001; Figure 4B). Although only a small fraction (<10%) of neurons were modulated by the first lick alone, ~30% were modulated by bouts of licking. Among neurons with bout-related firing modulations, a larger fraction (25.3%) were sensitive to bout onset compared to offset (6.8%; χ^2^ = 30.1, *p* << 0.001; Figure 4B). Examples of neurons with significant modulation around the first lick and at the beginning and end of licking bouts are shown in Figure 4C. These data indicate that the mPFC plays a role in monitoring the presence of reward, as well as tracking ongoing consummatory behaviors.

Principal Component Analysis revealed major variance in firing rates across the neuronal population around both the start and end of licking bouts (Figure 4D, far left, top panel). Neurons with strong loadings (reported as Z-scores) on the first Component showed increases or decreases in firing after the start of the bout, while neurons with strong loadings on the second Component increased or decreased firing acutely around the time of the bout onset (Figure 4D, to right of Components panel). Notably, the first Component was similar to the cumulative sum of the second Component, and vice versa (dashed lines in Figure 4D, far left, top panel), an effect that we also reported from the same type of analysis of lever-press related activity in a reaction-time task (Narayanan and Laubach, 2009). Such relationships were not observed in plots of the higher Components against the cumulative sums for any of the Components (not shown). This analysis indicated strong modulation of the neuronal population by the onset of licking bouts. That the cumulative sum of the second Component over time resembled the first Component indicates that the firing rates of neurons weighted strongly on Component 1 reflect the temporal summation of information from neurons weighted strongly on Component 2. Similar results were observed for the end of licking bouts (Figure 4D, panels at right).

**Figure 5:**
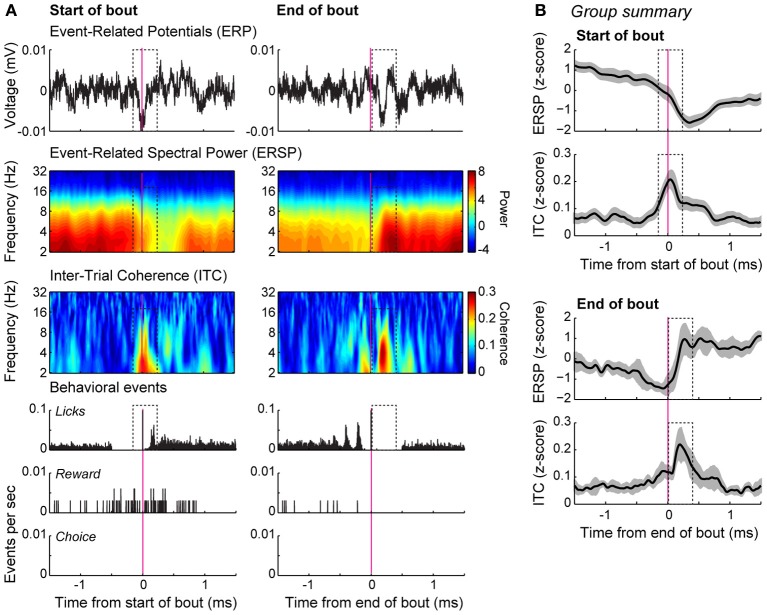
**Bout-related modulations of field potentials in the delayed alternation task. (A)** Spectral analysis of an example recording. The averaged event-related potentials (ERP) for the onset and offset of the bouts of licking are shown in the top row. Event-related spectral power (ERSP), depicted using a log scale over the frequencies, is shown in the second row. Inter-trial phase coherence (ITC) is shown in the bottom row. Coherence was increased around the initiation of the bouts (dashed box). Peri-event histograms for the behavioral events are shown below the spectral plots. **(B)** Group summary of the ERSP and ITC values for the onsets and offsets of the bouts are shown for the database of 54 field potentials recorded from 4 rats. The plots show the average level of power and coherence and 95% confidence intervals are indicated by the gray bands. The dashed boxes highlight the same temporal epochs that are highlighted in **(A)**, where there was consistent phase locking in the theta range bracketing the bouts of licks.

Network modulation by bouts of licks was examined using spectral analysis of field potentials. Power in the theta frequency range was reduced during the early portion of licking bouts for the field potential shown in Figure 5A (dashed box in panels in left column). The inter-trial phase coherence for this field potential was strongest around bout onset (Figure 5A, dashed box in panel in left column). This decrease in power and increase in coherence around bout onset were evident in the group summaries of event-related power and phase locking (Figure 5B, top panels). There was also an increase in theta power immediately following the cessation of licking, as well as an increase in theta-phase locking (Figure 5A, dashed box in right columns; Figure 5B bottom panels). These data together indicate a potential role for the mPFC in controlling the microstructure of licking by demonstrating strong network coherence when rats started and stopped bouts of licking.

### mPFC neurons are modulated around bouts of licking in the variable-interval licking procedure

**Figure 6:**
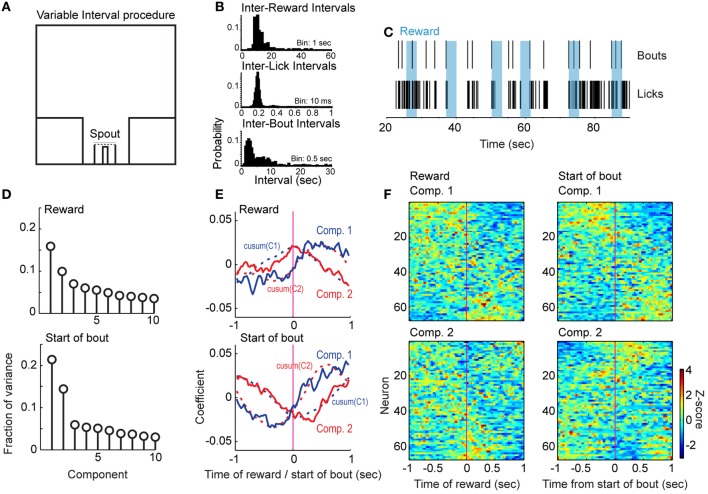
**Reward- and bout-related modulations in the variable-interval procedure. (A)** Design of the procedure. Rats responded at a reward port containing a contact-sensitive spout located between two barriers. They received water at a rate of 0.04 ml per s for 3 s approximately every 15 s. There was no access to other operanda in this task. **(B)** Examples of intervals between rewards (top), licks (middle), and bouts (bottom) from one recording session. **(C)** Raster display of licks and bouts over six rewards (blue) from the session in **(B)**. **(D)** Variance captured by the leading 10 Components from population analyses of firing rates in relation to the delivery of reward (top) and the bouts of licks (bottom). More variance was accounted for by the leading Components when the neuronal activity was aligned to the initiation of bouts than to the delivery of reward. **(E)** Temporal patterns of population activity around reward delivery (top) and the bouts of licks (bottom). The cumulative sums for each Component are also plotted as dashed lines. **(F)** Patterns in firing rates based on sorting average peri-reward and peri-burst histograms by the loadings for the first and second Components from the population analysis.

To examine whether reward and consummatory-related neuronal activity would be modulated in a task that should not depend upon mPFC for working memory function, we trained rats in a variable-interval licking procedure. Examples of inter-reward, inter-lick, and inter-bout intervals from a single session in one rat are shown in Figure 6B. Figure 6C shows the timing of reward delivery (blue boxes), bout onsets (top row), and individual lick events (bottom row) for six consecutive trials in the same animal and session.

In total, 67 neurons were recorded from the 3 rats included in this experiment. Principal Component Analysis around reward delivery and bout onsets (Figure 6D, top and bottom, respectively) revealed patterns of neuronal firing across the total population that were similar to those observed in the delayed alternation task. Neurons sorted by loading on these Components are shown in Figure 6F (reward delivery in left panels, bout onset in right panels). For bout onsets, the largest fraction of variance appeared to correspond to increased or reduced firing after the start of the bout (Figure 6E, lower panel and Figure 6F, top right panel). Neurons with strong loadings on the second Component showed increased or decreased firing immediately around the bout onset (Figure 6E, bottom panel and Figure 6F, lower right panel). As in the delayed alternation task, each of the first two Components was similar to the cumulative sum of the other Component (Figure 6E, dashed lines). These data support a role for mPFC in reward and consummatory processing, even when this region is not necessarily involved in the working memory processes with which it is often ascribed.

**Figure 7:**
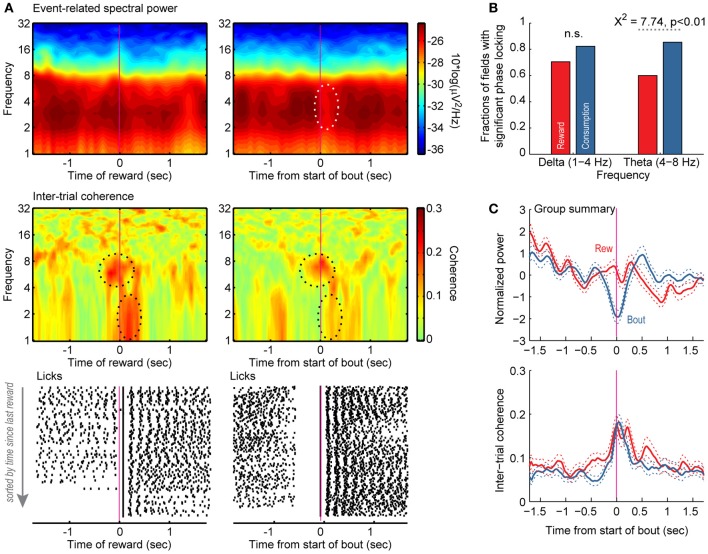
**Reward- and licking-related modulations of field potentials in the variable-interval procedure. (A)** Event-related spectral power (top) and inter-trial phase coherence (middle) are shown for a field potential and raster plots are shown for licking (bottom). The left plots are synchronized to the onset of reward. The right plots are synchronized to the onset of bouts of licks. Power was most prominent between 4 and 8 Hz, and there was a region near time 0 with significantly reduced power based on bootstrapping analysis (white dashed line). Two regions with significant phase locking are highlighted with black dashed lines, one between 4 and 8 Hz (theta) and the other between 1 and 3 Hz (delta). Significant phase locking in the range of theta occurred both when rats licked at the start of the reward period and when rats emitted bouts of licks at other times in the session. Significant delta phase locking was only detected when reward was delivered. **(B,C)** Group summaries for the fractions of fields with significant phase locking in the ranges of delta and theta and average event-related power and inter-trial coherence around the delivery of reward (red) and the onset of licking (blue).

Power in the range of 2–8 Hz—encompassing the delta (1–3 Hz) and theta (4–8 Hz) bands—was most prominent across the 55 field potentials recorded in the 3 rats from the variable-interval task (shown for a single field potential in one session in Figure 7A, top panel). Power across these frequencies was specifically reduced around bout onset and not reward delivery (Figure 7A, top panel). Significant theta phase-locking occurred both when reward was delivered and when rats emitted bouts of licks at other times in the session (Figure 7A, top dashed ellipse in middle panel). Significant delta phase-locking was only detected when reward was delivered (Figure 7A, bottom dashed ellipse in middle panel). Raster plots for licking activity corresponding to this field potential recording are shown in the bottom panels of Figure 7A. Large fractions of the recorded field potentials showed phase-locking around both reward and bout onset in the variable-interval task. There were no differences between the two events in the fractions of neurons with delta phase-locking (70.5% reward vs. 82.3% first lick, *p* > 0.05; Figure 7B, left). However, there were significantly more field potentials with significant theta-phase locking around bout onsets (85.4%) compared to reward delivery (60.0%; χ^2^ = 7.74, *p* < 0.01; Figure 7B). Across all recorded pfield potentials, power was specifically reduced around bout onsets (Figure 7C, top panel), but inter-trial coherence was evident around both behavioral events (Figure 7C, bottom panel). Analysis of field potentials within the mPFC during performance of the variable-interval task therefore indicate that this region is more generally involved in monitoring or controlling consummatory behaviors, and is not simply engaged during its recruitment in a working memory-dependent task.

### The rostral part of the mPFC is part of an anatomical network involved in licking behaviors

**Figure 8:**
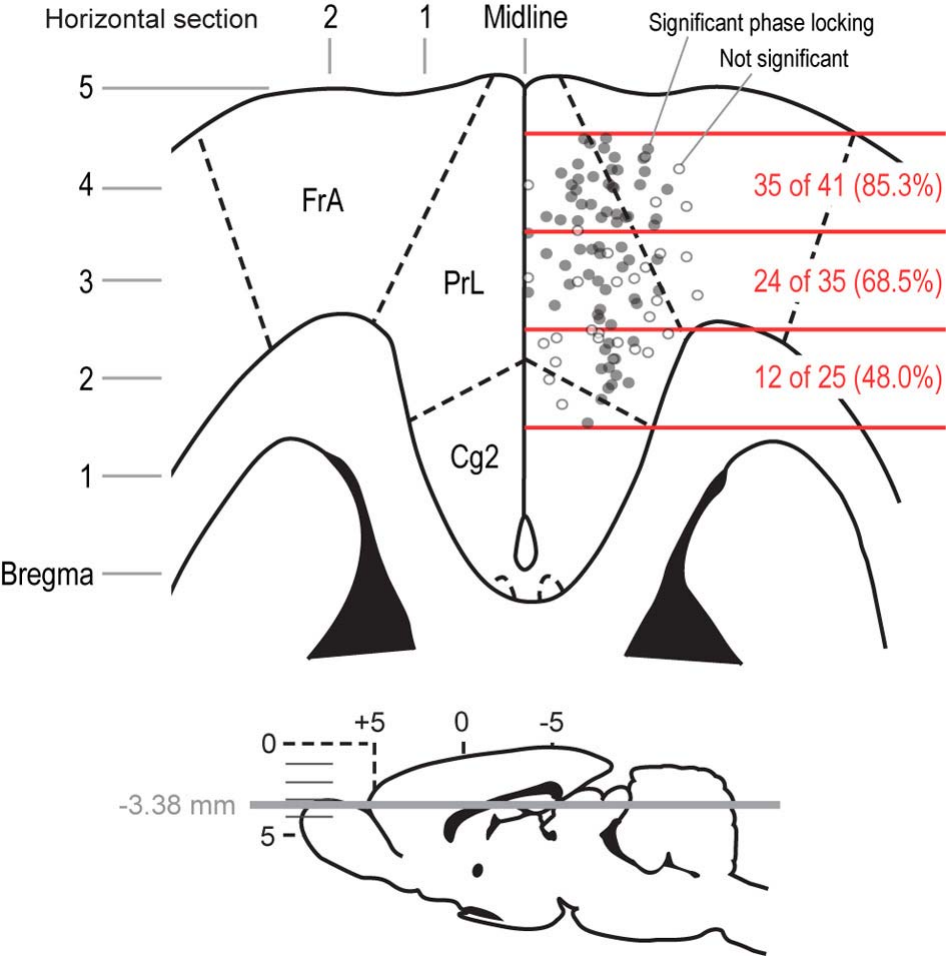
**Anatomical distribution of field potentials that showed significant phase locking around bouts of licks.** A horizontal section is shown to depict the locations of the recording sites for the field potentials that were analyzed in the delayed alternation and variable-interval tasks. The section represents the mPFC at a depth of 3.38 mm below the skull surface. Electrodes that had fields with significant phase locking at the initiation of licking are shown as filled gray dots. Electrodes that showed non-significant phase locking are shown as empty gray dots. The rostral-to-caudal span of the distribution of recording sites was split into three parts, which covered the peri-genual mPFC (caudal), the caudal prelimbic and adjacent agranular cortex (middle), and the rostral prelimbic and adjacent agranular cortex (rostral). The fractions of fields in these three regions that showed significant phase locking at the initiation of licking were significantly different by a Chi-square test (*p* < 0.001). That is, most sites in the rostral mPFC showed phase locking when rats emitted bouts of licks, and fewer sites in the middle and caudal mPFC showed such phase locking.

By anatomically mapping the electrode sites with theta phase-locking, we found that the highest proportion (85.3%) of sites with firing coherent in the 4–8 Hz range were in the most rostral portion of mPFC (Figure 8). This proportion dropped significantly in caudal sites (68.5% just caudal, and 48.0% near the corpus callosum). The difference in proportions was significant (Pearson's Chi-Squared test: χ^2^ = 10.5, *p* < 0.01). These data indicate that, as one would expect, the reward- and consummatory-related activity described above is most prominent in regions of mPFC with known projections to networks of brain regions involved in consumption.

## Discussion

This study examined the influences of rewards and consummatory behavior on neuronal activity in the mPFC during an operant-delayed alternation task and a variable-interval licking procedure. In both tasks, neuronal spike activity was modulated both by the presence of reward and by licking behavior. Licking-related responses included changes in firing rates around the onsets and offsets of bouts (clusters) of licks. These findings suggest that mPFC neurons participate in monitoring and/or controlling consummatory behavior. These changes in spike activity were accompanied by major changes in field potentials when rewards were delivered and rats emitted bouts of licks. Fluctuations in the range of theta (4–8 Hz) showed significant phase locking when rats initiated licking and when rats emitted bouts of licks. Anatomical reconstructions of the field potential recording sites showed that consumption-related activity was especially prominent in the rostral part of the pregenual mPFC, where licking-related activity has been previously reported by Petykó et al. (2009), and was less common in more caudal pregenual and peri-genual mPFC, as previously reported by Takenouchi et al. (1999). As discussed at length below, anatomical studies have found that the rostral mPFC is interconnected with the agranular insular cortex (Gabbott et al., 2003) and projects to feeding-related centers in the hypothalamus, midbrain, and brainstem (e.g., Floyd et al., 2000, 2001). Our findings suggest that the mPFC serves to monitor and control consummatory behavior through these connections.

### Neuronal spike activity in the mPFC is modulated around reward delivery and consumption

In the delayed alternation task, many neurons in the mPFC showed changes in firing rates around the onset of rewarding fluid, licking, or both of these events (Figure 3B). The total fraction of reward- and consumption-modulated neurons (~50%) was greater than the fractions of cells modulated around other events in the task, e.g., ~35% of neurons modulated around the spatial choice in the version of the task used in Horst and Laubach (2012) and 20–40% of neurons (measured over a series of sliding windows) from young rats that were modulated by the spatial choice in an alternate design used in Caetano et al. (2012a), in which rewards were given in each choice port. As a population, mPFC neurons showed major changes in activity that appeared to be time-locked to the onset of reward delivery. As in the single neuron analyses summarized above, the fraction of variance accounted for by the two leading Principal Components around the time of reward collection (52.9%) was greater than that associated with the spatial response (47.6%) that triggered access to the reward (Figure 1D). Similar common fluctuations in spike activity were also found in the variable-interval licking task (Figures 6D,E), suggesting that the changes in neuronal activity accounted for by these population functions did not necessarily depend on, or arise due to, the intervening spatially directed locomotor behaviors that occurred in the delayed alternation task.

By delaying the onset of reward relative to the onset of licking in the delayed alternation task, we were able to show in Figure 3 that reward-related network activity (i.e., field potentials) was driven more by the initiation of licking and not the delivery of reward. Neurons showed phasic increases or decreases in spiking activity around reward onset, marking licking onset as an important behavioral event. The relationship between these patterns of activity around the bouts of licks, as measured by the cumulative sum analysis in Figure 4D, is suggestive of temporal integration within the neuronal population. This finding is similar to what we have previously found in mPFC for neuronal activity associated with temporally constrained lever pressing (Narayanan and Laubach, 2009). Such integration could serve to encode the animal's “action state” and allow for encoding the time spent in a given state, based on the dynamically changing nature of the population functions.

We found it interesting that the patterns of population activity in the delayed alternation and variable-interval tasks were similar, especially as there was no locomotor activity preceding reward delivery and engagement in licking in the variable-interval task. These findings might suggest that mPFC neurons encode a consumption specific “action state,” as discussed above, or the task context, i.e., the “reward period.” Such a contextual encoding would reflect the phase of the task in which the rat is currently engaged and might be crucial for the ability to transition between phases of the task, a process that appears to be reduced in aging (Caetano et al., 2012a). Support for this context-based interpretation also comes from a study by Mulder et al. (2003) who reported that mPFC neurons are modulated by the phases of a go/nogo task. In that task, neurons showed depressed activity between the time of stimulus onset and reward delivery on go trials and elevated firing between the beginning of reward collection and the next presentation of the stimulus. This activity is quite similar to the reward period activity in the delayed alternation task that is reported here. Instead of waiting for a cue, rats were free to initiate the response sequence at their own pace, but the responses themselves likely acted as cues for subsequent responses. Additional experimental support for this hypothesis is provided from recordings in the ACC, which revealed distinct network states that varied specifically with task epoch (Lapish et al., 2008). Based on these independent findings, it would seem that afferent projections to the mPFC provide information about temporal and spatial aspects of the animal's current behavioral situation (“task context”) which can then be used to determine the appropriate motor output based upon previous experiences in a similar context. This is consistent with the conclusions from a recent review of mPFC function (Euston et al., 2012).

### Field potentials in mPFC were phase locked to the initiation of reward consumption

Field potential recordings were analyzed using time-frequency methods to determine if these signals reflected reward expectation [as proposed by (Van Wingerden et al., 2010) for the orbitofrontal cortex (OFC)] or the animals' consumption of the reward [(as proposed by (Gutierrez et al., 2006) for the OFC)]. Phase locking was specific to the delta (1–4 Hz) and theta (4–8 Hz) frequency bands, and was predominantly observed around the onset of licking and not reward delivery. Phase locking around the initiation of licking was found both in the delayed alternation and variable-interval tasks. The time points with phase locking were associated with changes in spectral power, especially when rats transitioned from traveling to the spout in the delayed alternation task, and with changes in population spike activity (as revealed by PCA).

To further examine this issue, we used standard methods for the temporal analysis of licking (i.e., microstructure analysis as used by (Gutierrez et al., 2006) and many other studies) and assessed changes in neuronal firing rates around these bouts of licks. We removed bouts that were coincident with the onset of reward, and thus were able to examine neuronal modulations that were not associated with receipt of the reward. This analysis found that ~25% of neurons in mPFC were modulated around the bouts of licks in the delayed alternation task (Figure 4B) and many field potentials showed phase locking around these events (Figure 5). These findings suggest that theta-range fluctuations in the mPFC primarily reflect consummatory behaviors (Gutierrez et al., 2006), not reward expectation (Van Wingerden et al., 2010).

### Functional specialization of the rostral mPFC for processing reward-related information

Motivated by the finding that not all field potentials showed evidence for phase locking around the initiation of bouts of licking, we made maps of the recording sites and plotted electrodes that showed significant and non-significant levels of phase locking (Figure 8). Two interesting observations were made following this procedure. First, not all electrodes within a given region of mPFC showed phase locking at the time of bouts of licks, a finding that suggests that phase locking was not due to volume conduction from other brain regions. Moreover, the local heterogeneity with regard to bout-related phase locking suggests that differences in the local microcircuit or the local connectivity of the mPFC may contribute to the processing of consummatory behavior. Second, field potentials with significant phase locking at the onset of licking were concentrated in the rostral portion of mPFC, a region with prominent inter-connections with the agranular insular cortex (Gabbott et al., 2003), a region that is responsive to gustatory and visceral sensory signals, and prominent projections to feeding-related centers in the hypothalamus (Floyd et al., 2001), peri-acqueductal gray (Floyd et al., 2000), and other brainstem areas (Gabbott et al., 2005). Based on our findings, and these anatomical studies, we propose that the rostral part of the mPFC, especially the rostral prelimbic area, is specialized for controlling consumption.

### Relevance of consummatory activity in mPFC for learning and natural foraging

Changes in reward-related signals during learning and periods of behavioral uncertainty (e.g., reversal learning) could serve to bias animals to engage in reward-seeking behaviors, as has recently been reported in a study of reversal learning (Kimchi and Laubach, 2009) and shown to occur following inactivation of the mPFC (Ishikawa et al., 2008a,b). Knowledge about reward and its relationship to behavioral responses could be acquired by monitoring consummatory-related neuronal activity, as has been shown in a series of studies in another prefrontal region. Gutierrez et al. (2006); Gutierrez et al. (2010) demonstrated that, in addition to distinguishing between natural rewards, many neurons in the OFC-tracked licking behavior *per se*. Inactivation of OFC disrupted the microstructure of licking activity (Gutierrez et al., 2006). Furthermore, there was synchronous activity among neurons in OFC, insular cortex, amygdala, and nucleus accumbens during licking and the activity of these neurons increased with learning and discriminated between different tastants (Gutierrez et al., 2010). These data suggest that a distributed set of forebrain areas is engaged by consummatory behavior, and that there is a refinement of consumption-related activity during learning.

There is an interesting convergence of spatial- and reward-related processing in the mPFC, which might reflect the evolutionary significance of the brain region. Early mammals had to forage in dynamic environments. They needed to determine when to forage (based on measures such as cloud cover and ground temperature, factors that still control foraging behavior in modern rodents: e.g., Orrock et al., 2004). They needed to remember where they recently found food and to be able to predict where food is likely to be in the future. Finally, they needed to be able to determine if foods that are found have caloric value and are worth the costs of foraging. All of these foraging variables would presumably need to be encoded using some type of long-term memory and retrieved and dynamically decoded using some type of short-term (or working) memory. Embedding these functions in the brain may occurred by taking advantage of the recurrent circuits and highly plastic synapses that are found in the hippocampus and prefrontal cortex.

These systems converge onto the ventral striatum, a region that has been shown to encode the palatability of foods (Taha and Fields, 2005), the occurrence of meals during natural feeding behavior (Tellez et al., 2012), and to control food-seeking behavior through its connections with the dopaminergic (Baldo and Kelley, 2007), opioid (Kelley et al., 2005), and hypothalamic peptide (Sears et al., 2010) systems. [A recent study has implicated similar opioid control of feeding directly within the mPFC (Mena et al., 2011).] We propose that major progress will be made on understanding the neuronal basis of foraging by focusing on interconnections between cortical, hippocampal, and amygdalar regions that send convergent projections to the anterior part of the ventral striatum. Animals engage in unregulated food-seeking behaviors when this part of the striatum is reversibly inactivated (Sears et al., 2010), and this striatal region receives projections from the mPFC, agranular insular cortex, hippocampal formation, and the amygdala (see Groenewegen and Trimble, 2007, for review). Convergent activity among these distributed brain regions onto the ventral striatum could enable learning (as proposed in Laubach, 2005) about the values of foods and the contexts in which food is consumed.

### Conflict of interest statement

The authors declare that the research was conducted in the absence of any commercial or financial relationships that could be construed as a potential conflict of interest.
